# Potential therapeutic approaches for modulating expression and accumulation of defective lamin A in laminopathies and age-related diseases

**DOI:** 10.1007/s00109-012-0962-4

**Published:** 2012-10-23

**Authors:** Alex Zhavoronkov, Zeljka Smit-McBride, Kieran J. Guinan, Maria Litovchenko, Alexey Moskalev

**Affiliations:** 1 Bioinformatics and Medical Information Technology Laboratory, Center for Pediatric Hematology, Oncology and Immunology, Moscow, 119296 Russia; 2 The Biogerontology Research Foundation, Reading, UK; 3 Department of Ophthalmology and Vision Science, School of Medicine, University of California at Davis, Davis, CA 95616 USA; 4 BioAtlantis Ltd., Kerry Technology Park, Tralee, County Kerry Ireland; 5 Laboratory of Molecular Radiobiology and Gerontology, Institute of Biology, Komi Science Center of Russian Academy of Sciences, Syktyvkar, 167982 Russia

**Keywords:** Lamin A, Progeria, Laminopathies, Age-related diseases, Aging

## Abstract

Scientific understanding of the genetic components of aging has increased in recent years, with several genes being identified as playing roles in the aging process and, potentially, longevity. In particular, genes encoding components of the nuclear lamina in eukaryotes have been increasingly well characterized, owing in part to their clinical significance in age-related diseases. This review focuses on one such gene, which encodes lamin A, a key component of the nuclear lamina. Genetic variation in this gene can give rise to lethal, early-onset diseases known as laminopathies. Here, we analyze the literature and conduct computational analyses of lamin A signaling and intracellular interactions in order to examine potential mechanisms for altering or slowing down aberrant *Lamin A* expression and/or for restoring the ratio of normal to aberrant lamin A. The ultimate goal of such studies is to ameliorate or combat laminopathies and related diseases of aging, and we provide a discussion of current approaches in this review.

## Introduction

The nuclear lamina is an array of intermediate filament proteins inside the nucleus of eukaryotic cells, which supports the structure of the nucleus, including its shape and mechanical stability [1, 2]. In addition, it serves as a scaffold for the attachment of DNA–protein complexes that regulate both eu- and heterochromatin histone modifications [3]. The nuclear lamina is involved in the regulation of many key biological processes, including DNA replication, transcription, cell cycle progression, and chromatin organization [2]. Given the central role of the nuclear lamina in such a wide range of essential processes, it is not surprising that alterations in the structure can have a significant impact on normal cellular function, and in some cases can give rise to disease and even mortality within affected organisms.

Maintenance of the nuclear lamina is essential for most eukaryotic life forms, and requires the presence of an array of specific proteins that are highly conserved evolutionarily, both in terms of their structure and function. In particular, major functional components of the nuclear lamina are fibrous proteins known as nuclear lamins, which support this structure through interactions with specific membrane-associated proteins. Lamins are highly conserved evolutionarily, being represented in all examined metazoan life forms; thus, their essential functions likely ensure survival across a broad range of species [4]. Mutations within lamin genes and subsequent alterations in the structure and function of the proteins they encode can give rise to a broad range of diseases known as laminopathies. Such diseases are characterized by a broad range of severe clinical symptoms and complications, with some causing mortality early in life. Numerous laminopathies have been identified in humans during the last decade, and have been linked to several types of mutations in causative loci, both within lamin genes themselves and in genes encoding lamin-binding proteins. Laminopathies include Emery–Dreifuss muscular dystrophy (MIM 181350), dilated cardiomyopathy (MIM 115200), familial partial lipodystrophy (MIM 151660), Charcot–Marie–Tooth disorder type 2B1 (MIM 605588), Greenberg skeletal dysplasia (MIM 215140), limb girdle muscular dystrophy Type 1B (MIM 159001) and mandibuloacral dysplasia with type A lipodystrophy (MIM 248370). The molecular mechanisms by which lamins contribute to these diseases have become increasingly understood in recent years, particularly in terms of the genetic mutations and effects therein on both gene expression and protein structure and function.

## Lamin A processing, mutations, and role in diseases

Lamins can be categorized as either A type (lamins A and C) or B type (lamins B1 and B2). In humans, A-type lamins are encoded by a single gene—*LMNA* (*Entrez Gene ID*: *4000*)—located on chromosome 1q21.2, while B-type lamins are encoded by two genes—*LMNB1* and *LMNB2* (*Entrez Gene ID*: *4001 and 84823*)—located on chromosomes 5q23.2 and 19p13.3, respectively. The processes involved in the expression of lamin genes and their translation and processing into mature and functional proteins include a series of specific and essential steps, alterations to which can impact the essential molecular and cellular functions of these proteins. In the case of LMNA, one essential step in protein biosynthesis and maturation is farnesylation at the C-terminus by the enzyme farnesyltransferase [5]. This posttranslational modification plays a role in targeting prelamin A to the inner nuclear membrane. Farnesylation is followed by several steps involving the endoproteolytic cleavage of the last three amino acids by zinc metallopeptidase ZMPSTE24, carboxymethylation of the C-terminal cysteine by ICMT methyltransferase, and proteolytic removal of the last 18 amino acids by ZMPSTE24, resulting in the removal of the farnesyl tail on the C-terminus [6, 7]. Mature lamin A is then released from its membrane anchor, which allows it to be properly positioned in the nuclear scaffold. Factors that interfere with these steps in such a way as to affect lamin maturation can have negative effects on nuclear lamin and can ultimately lead to an array of downstream effects, detrimental to cellular health and in some cases, longevity.

Within the *LMNA* gene alone, over 400 different point mutations have been identified, many of which are underlying causes of laminopathies [8, 9], including restrictive dermopathy (MIM 275210) and Hutchinson–Gilford progeria syndrome (HGPS; MIM 176670). HGPS presents as a broad range of clinical features, which most notably include accelerated aging [10]. HGPS is caused by mutations in the *LMNA* gene, the most well-known of which is a de novo heterozygous point mutation in position 1824C > T (G608G) [11]. While the *G608G* mutation does not cause any change in the encoded amino acid, it does activate a cryptic splice donor site in exon 11 of the *LMNA* gene. Consequently, a splice variant of *Prelamin A* mRNA is generated with an internal deletion of 150 base pairs [11]. These transcripts are translated into progerin, the truncated form of the lamin A protein, with a 50 amino acid internal deletion near the C-terminus [11]. The internal deletion eliminates the essential endoprotease ZMPSTE24 recognition site, resulting in progerin remaining permanently farnesylated and anchored to the nuclear membrane [11, 12]. The accumulation of progerin in cells of patients carrying the *G608G* mutation severely impacts the structure of the nuclear lamina, culminating in the cellular and disease phenotypes characteristic of HGPS.

Severe forms of progeria also occur due to a number of other mutations in *LMNA*, such as 1821 G > A and 1,968 G > A, mutations associated with increased ratios of progerin to normal, wild-type protein [13]. An extremely severe case of neonatal progeria in which death occurs within the first year of life has recently been found to be associated with heterozygosity (1,821 G > A). Examination of patient fibroblasts demonstrates an increased ratio of progerin to lamin A, relative to those levels typically observed in HGPS, suggesting that disease severity may be determined in part by the ratio of the farnesylated protein to mature lamin A [13, 14].

The hallmarks of progeria and its characteristic phenotypes are broadly associated with alterations in the production of progerin relative to mature lamin A, imbalances that directly impact key biological processes occurring at both the genetic and cellular levels. Progerin is observed to accumulate in all tissues of HGPS patients, acting as a dominant-negative protein that significantly modifies the structure of the nuclear lamina [15]. The cellular phenotype of HGPS patients includes nuclear blebbing, thinning of the nuclear lamina, loss of peripheral heterochromatin, and clustering of nuclear pores [16]. Accumulation of progerin in HGPS as nucleoplasmic aggregates leads to inhibition of the transport of several factors that play key roles in the functioning of the nucleus [15]. Examination of fibroblast cells from patients with HGPS demonstrates deficiencies in histone modification, alterations in gene expression, delays in the response to DNA damage, disturbances of mitosis, and cytokinesis, abnormalities in chromosome segregation and increases in the occurrence of binucleated cells [9]. The hallmarks and clinical features of HGPS are therefore deeply rooted in alterations taking place at genetic and protein biosynthesis levels and, in turn, those subsequent changes that negatively impact key biological processes further downstream. While there are significant components to HGPS that are associated with aging, disease pathogenesis, and progression is likely to involve several factors not exclusive to the aging process. While many tissues in HGPS patients exhibit phenotypes associated with accelerated aging, not all tissues are typically affected (reviewed by [17]). In addition, HGPS may not be viewed exclusively as a disease of accelerated aging, given that certain aspects of the disease are not typically associated with normal aging; for example, the presence of clavicular agenesis. Significant features of HGPS that are associated with normal aging include increases in DNA damage, defects in DNA repair, alterations in telomeric dynamics, and increases in cell proliferation, senescence, and tissue homeostasis (reviewed by [17] and references therein). In this respect, HGPS may be viewed as a disease that substantially resembles premature aging, but does not include all aspects of it, and is segmental in nature.

## The significance of LMNA in human health and longevity

Several genes have been identified in recent years as influencing the aging process and possibly longevity [18, 19]. The potential significance of the *LMNA* gene in human health and its potential contribution to susceptibility to many common diseases is also becoming increasingly appreciated. In particular, Scaffidi et al. demonstrate that the molecular mechanism that underlies HGPS also takes place in normal cells at a lower rate [20]. The nuclei of cells of normal-aged individuals exhibit defects similar to those of cells of HGPS patients, including changes in histone modification and increased levels of DNA damage. Age-dependent defects in the nuclei of cells of healthy individuals are caused by infrequent use of the same cryptic splice site of *Lamin A*, whose constitutive activation generates a *Progerin* transcript [20]. The over-expression of normal *Prelamin A* can lead to growth defects in human vascular smooth muscle cells [21], similar to those changes observed in cells producing *Progerin* [22]. Cytotoxicity can also be induced by a minor increase in the steady-state level of one or more intermediate products of *Prelamin A* processing [12, 22].

Recent years have shown extensive investigation of the potential contribution of genetic variability within lamin genes to disease susceptibility. Disease-association studies including SNPs at lamin loci, have implicated metabolic syndrome, dislipidemia, type-II diabetes, obesity, polycystic ovary syndrome, arterial stiffness, and vascular disease [23–35]. In addition, there is some evidence for the potential influence of genetic variation at *LMNA* on human longevity and age-related diseases [36–38]. Findings from these studies have been variable, with the majority focusing on the 1908C > T; rs4641 *LMNA* SNP. rs4641 has been found in several cases to be significantly associated with disease susceptibility and related conditions across a number of ethnically diverse population cohorts for type II diabetes and related diseases [23, 24, 26–28, 31]. The rs4641 SNP is a silent C > T substitution occurring at exon 10 of the *LMNA* gene, the exon in which alternative splicing gives rise to mRNAs that code for either *Prelamin A* or *Lamin C* [39]. The mechanism by which this SNP alters the *LMNA* gene product and phenotype to potentially influence susceptibility to these diseases is unknown. However, recent evidence suggests that the C and T alleles of rs4641 are associated with differential gene expression phenotypes, with the C allele associated with increased levels of transcripts of *Lamin A* and *Lamin C* relative to those detected for the T allele [40]. While this study demonstrates that differential, allele-specific expression is present at the *LMNA* locus in HGPS, it is unclear whether or not such variability is directly associated with the rs4641 SNP or if it is rather associated with other variants located within the same haplotype block. In light of these studies, the relevance of genetic variation at the *LMNA* locus to more common diseases affecting populations at large may be significant. Interestingly, the rs4641 SNP is represented in all populations that have been examined in the HapMap project to date, with the minor ‘T’ allele represented at levels ranging from between 5 and 10 % in African populations, 20–25 % in Europeans and 23–32 % in South and East Asian populations (www.hapmap.org) [41]. Elucidating the role of this relatively common SNP in disease pathogenesis, longevity, or related diseases, therefore, may have broad significance. However, in-depth examination of linkage disequilibrium between rs4641 and other functional SNPs is required to delineate the role of this *LMNA* SNP in human diseases, metabolic-related, age-related, or otherwise.

Given the evidence that genetic variation at *LMNA* contributes to both laminopathies and more common human diseases, the identification of methods that can therapeutically alter *LMNA* structure and restore a healthy homeostatic balance of aberrant/normal *LMNA*, warrants further investigation. A multifaceted approach is required to increase knowledge in this area and further elucidate the functional relevance and complex characteristics of lamins both in terms of their expression and functional interactions. In this way, interventions may be designed and developed to intervene, treat, and ameliorate symptoms of human diseases, particularly those associated with aging.

## Targeting LMNA and associated diseases: a multifaceted approach

Combating the broad range of consequences associated with missplicing lamins and altering their gene expression levels requires a multifaceted approach, which includes targeting components at the genetic level as well as targeting components downstream cell signaling and cellular-level processes. Furthermore, unraveling the underlying complexity at each of these levels and, in turn, targeting specific processes therein to treat or ameliorate disease symptoms, requires (a) an in-depth knowledge of the molecular interactions between nuclear lamins and other proteins and cellular events, (b) in vitro and in vivo studies demonstrating effectiveness of the treatment and validating such interventions, and (c) development and refinement of techniques to manage, limit, and potentially reverse damage that has already been incurred in patients. Here, we perform an analysis of the existing literature and published data sets, with the goal of identifying novel targets for treating laminopathies and associated diseases. In turn, we hope that these findings may provide a basis for future experimental design, interpretation of results, and refinement of methods aimed at tackling severe laminopathies and other age-related diseases.

## Computational analysis of LMNA signaling and intracellular interactions

In this study, we utilized literature searches of the NIH’s PubMed database in order to examine pathways that regulate *LMNA* expression. This was accomplished by using the following keywords during searches: gene expression regulation *Lamin* A/C or *Progerin*, and progeria. In order to visualize the molecular interactions between nuclear lamins and other proteins and identify novel targets, the Ingenuity Pathway Analysis (IPA, Ingenuity© Systems, www.ingenuity.com) software and knowledgebase and its pathway designer graphical module were utilized. IPA provided graphical representations of network interactions of the LMNA protein with molecules involved in signal transduction pathways and other intracellular regulatory networks. Data used to generate pathways and interaction networks in IPA are compiled from interactions validated in multiple model organisms from peer-reviewed journals by a team of IPA scientists. Advantages of this software tool include: (a) each connection displayed on a graph is documented by a peer-reviewed article, which can be examined by clicking on the relevant connection and (b) the Pathway Designer module contains “cell art” elements which can be used to graphically display connection locations (nucleus, mitochondria, cellular membrane, etc.).

**Fig. 1:**
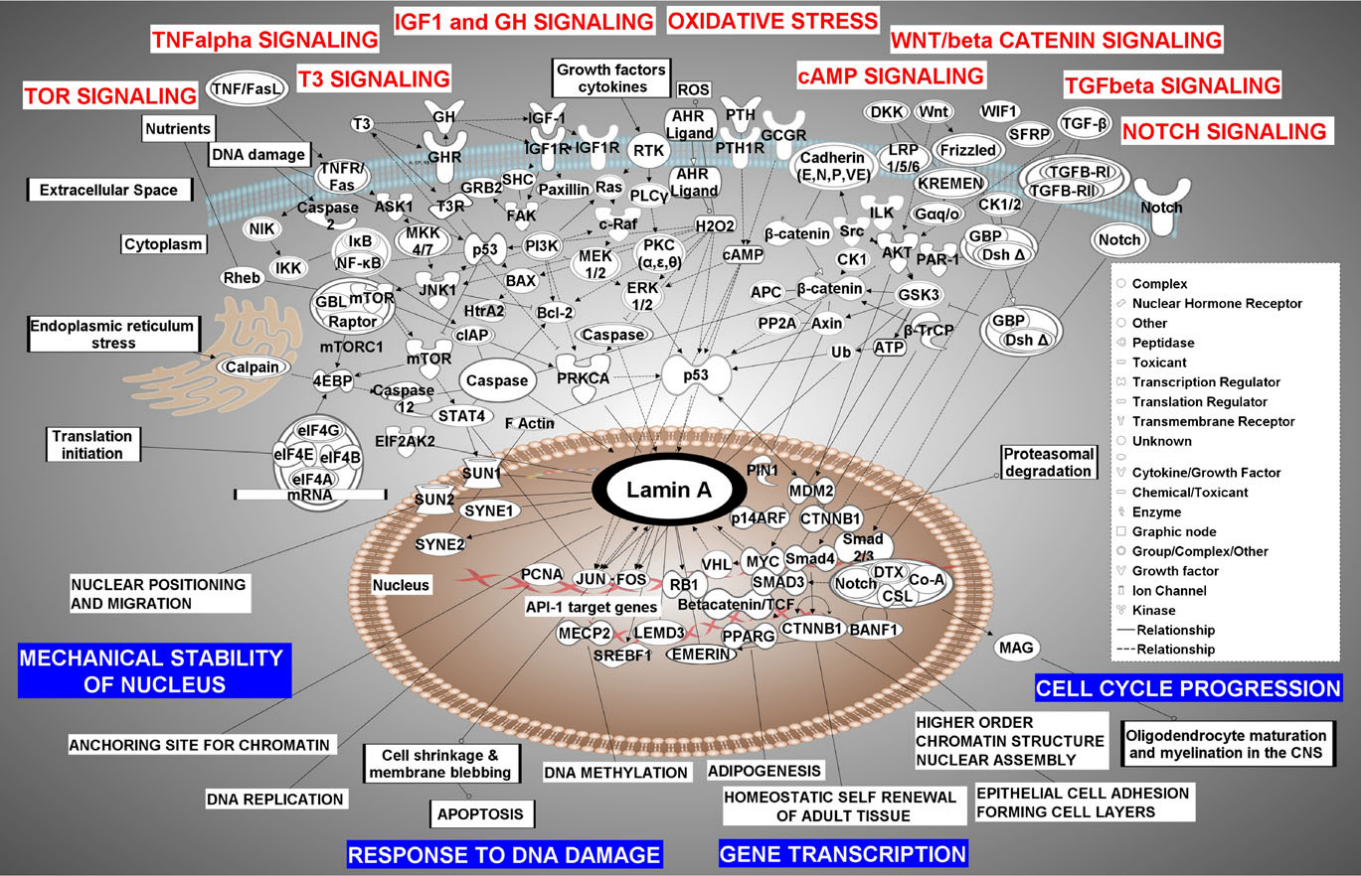
Key signaling pathways upstream of LMNA, which regulate its expression (listed mainly on the *top* of the figures), and the main molecules that interact with lamin A/C protein (listed mainly in the nucleus). Labels denoting physiological processes in the cell are derived partially from the IPA listing under category “Top Functions and Diseases”: *Mechanical Stability of the* *Nucleus*, *Response to DNA Damage*, *Gene Transcription*, *Cell Cycle Progression*. The meanings of the molecular symbols are described in the *figure legend*, which is part of the figure. *Lines* represent interactions between the molecules. They can have *arrows*, *solid lines* or nothing at the end, which represent directional action, inhibitory action, or just binding of two molecules, respectively. *Red crossing lines* in the nucleus represent a DNA double helix. Entrez Protein Names and their *symbols* used in figures, are in *parenthesis*: Catenin (*cadherin-associated protein*), beta 1, 88 kDa (β*-catenin*), beta-transducin repeat containing (*β-TrCP*), eukaryotic translation initiation factor 4E binding protein 1 (*eIF4EBP*), AHR ligand, aromatic hydrocarbon (*AHR Ligand*), protein kinase B/Akt (*AKT*), Adenomatous polyposis coli, APC (*APC*), mitogen-activated protein kinase kinase kinase 5 (*ASK1*), adenosine 5′-triphosphate (*ATP*), axin 1 (Axin), barrier to autointegration factor 1 (*BANF1*), BCL2-associated X protein (*BAX*), B cell CLL/lymphoma 2 (*Bcl-2*), beta-catenin-LEF/TCF (*Betacatenin*/*TCF*), V-raf-1 murine leukemia viral oncogene homolog 1(*c-Raf*), cadherin (*E*, *N*, *P*, *VE*), calcium-dependent protease, M calpain (*Calpain*), CASPASE-1 (*Caspase*), interleukin 1 converting enzyme (*ICE*), caspase 12 apoptosis-related cysteine peptidases (*Caspases 2*,*3*,*6* and *12*), casein kinase I (*CKI*), cyclic AMP (*cAMP*), eukaryotic translation initiation factor 2-alpha kinase 2 (*EIF2AK2*), eukaryotic translation initiation factors (*eIF 4A*, *4B*, *4G,* and *4E*), emerin (*EMD*), p42/p44 MAP kinase (*ERK1*/*2*), filamentous actin (*F Actin*), PTK2 protein tyrosine kinase 2 (*FAK*), FBJ murine osteosarcoma viral oncogene homolog (*FOS*), frizzled (*FZ*, *FZD*), MTOR-associated protein, LST8 homolog (*S. cerevisiae*) (*GBL*), frequently rearranged in advanced T cell lymphomas (*GBP*), glucagon receptor (*GCGR*), growth hormone receptor (*GHR*), growth factor receptor-bound protein 2 (*GRB2*), growth hormone (*GH*), glycogen synthase kinase 3 (*GSK3*), hydrogen peroxide (*H*_*2*_ *O*_*2*_), HtrA serine peptidase 2 (*HtrA2*), I KAPPA B (*IκB*), insulin-like growth factor 1 (somatomedin C) (*IGF-1*), insulin-like growth factor 1 receptor (*IGF1R*), I kappa β-NF-kappa β (*Iκβ-NFκβ*), integrin-linked kinase (*ILK*), mitogen-activated protein kinase 8 (*JNK1*), Jun proto-oncogene (*JUN*) lamin A/C (*LMNA*), LEM domain containing 3 (*LEMD3*), myelin-associated glycoprotein (*MAG*), p53 binding protein homolog (mouse) (*MDM2*), methyl CpG-binding protein 2 (Rett syndrome) (*MECP2*), MAP kinases (*MEK1*/*2*, *MKK4*/*7*, *MKK4*/*7*), mechanistic target of rapamycin (*serine*/*threonine kinase*) mTORC1 (*mTOR*), V-myc myelocytomatosis viral oncogene homolog (avian) (*MYC*), NF-KAPPA B(*NF-kB*), cyclin-dependent kinase inhibitor 2A (melanoma, p16, inhibits CDK4) (*p14ARF*), tumor protein p53 (*p53*, *TP53*), microtubule affinity-regulating kinase 2 (*MAP*), proliferating cell nuclear antigen (*PCNA*), 1-phosphatidylinositol 3-kinase (*PI3K*), peptidylprolyl cis/trans isomerase, NIMA-interacting 1 (*PIN1*), protein kinase C (*PKC*), phospholipase C gamma, (*PLCG*), protein phosphatase type2a (*PP2A*), peroxisome proliferator-activated receptor gamma (*PPARG*), protein kinase C, alpha (*PRKCA*), parathyroid hormone (*PTH*), parathyroid hormone 1 receptor (*PTH1R*), regulatory associated protein of MTOR, complex 1″ (*Raptor*), p21 Ras (*Ras*), retinoblastoma 1 (*RB1*), Ras homolog enriched in brain (*Rheb*), (Src homology 2 domain containing)-transforming protein 1 (*SHC*), SMAD family members (*Smad3* and *Smad4*), V-src sarcoma (*Schmidt-Ruppin A-2*) viral oncogene homolog (avian) (*Src*), sterol regulatory element-binding transcription factor 1 (*SREBF1*), signal transducer and activator of transcription 4 (*STAT4*), Sad1 and UNC84 domain containing 1 (*SUN1*), Sad1 and UNC84 domain containing 2 (*SUN2*), spectrin repeat containing, nuclear envelope 1 (*SYNE1*), spectrin repeat containing, nuclear envelope 2 (*SYNE2*), TGF beta (*Tgfβ*), thyroid hormone (*T3*), thyroid hormone receptor (*TR*), polyubiquitin (*Ub*), von Hippel–Lindau tumor suppressor (*VHL*), WNT inhibitory factor 1 (*WIF1*)

Searches did not identify signaling or metabolic pathways in IPA that center on LMNA. However, LMNA was found to be part of a canonical Apoptosis Signaling pathway as a target of caspase 6. The LMNA Interactome generated through this analysis displays 110 direct molecular interactions with LMNA, with all the known molecules that LMNA/lamin A interacts with, including proteins, protein modifiers, small molecules, and microRNAs. Some of these molecules are members of other signal transduction pathways, and therefore represent a bridge between LMNA and these pathways. The most important signal transduction pathways that target/affect LMNA are shown in Fig. 1. Data shown in this figure was generated by combining information from several individual canonical pathways, the LMNA Interactome, and additional information from the published literature, and then repeating several iterations of this process to reach a final model. As shown in Fig. 1, WNT/beta-catenin, TGF beta, Notch, and PI3K represent the key signaling pathways upstream of *LMNA*, which likely regulate its expression. The main molecules that interact with the lamin A protein, and have genetic correlates with some of the laminopathies we have discussed here, are mainly found in the nucleus, including Sun1 and Sun2, whose potential roles in HGPS disorder have been investigated recently [42]. Many of the identified signaling pathways and molecules that interact with lamin A are known to exert effects on nuclear lamins by altering their expression levels. To visualize gene expression regulatory points that potentially may be targeted for intervention, the Pathway Designer module was applied to the data presented in Fig. 1. Potential interventions might include following mechanisms: transcription, splicing, translation, posttranslational modification, and degradation via autophagy. Using the overlay function of IPA, this figure was overlaid with a number of pharmaceuticals and drugs that may be used to target key proteins of any given biological process. While a number of drugs were identified via the IPA database, more detailed lists of drugs and agents that may be applicable are listed in Tables 1, 2, 3, 4 and 5 generated through PubMed searches using relevant keywords. To expand the literature search and identify additional known drugs and experimental compounds, as well as their side effects, potentially acting on elements of the pathways involving LMNA gene, we employed a manually curated proprietary database (MetaCore™, GeneGo), and the MetaCore pathway analysis software.

**Table 1:** Notch signaling inhibitors

Compound	Mechanism of action	References	Side effects	SE references
SEL-10	Ubiquitin-mediated protein degradation	[119]	N/A	–
L-658,458 (transition state mimic)	γ-Secretase-inhibitors	[120]	N/A	–
*N*-[*N*-(3,5-difluorophenacetyl)-l-alanyl]-*S*-phenylglycine t-butyl ester (DAPT)	γ-Secretase-inhibitors	[120]	N/A, preclinical trials	[121]
IL-X (cbz-IL-CHO)	γ-Secretase-inhibitors	[122]	Neutropenia, injection-site reactions	[123]
WPE III-31-C	γ-Secretase-inhibitors	[124]	N/A	–
Compound E	γ-Secretase-inhibitors	[124]	N/A	–
Sulfonamide	γ-Secretase-inhibitors	[124]	Itching, burning, skin rash, redness, swelling, urinary tract disorders, haemopoietic disorders, porphyria, and hypersensitivity reactions	[121, 125]
JLK6	γ-Secretase-inhibitors	[124]	N/A	–
Sulindac sulfide	γ-Secretase-inhibitors	[124]	Stupor, diminished urine output and hypotension	Information from the manufacturer
Indomethacin	γ-Secretase-inhibitors	[124]	Nausea, dyspepsia, headache, dizziness	[121, 126]
Estrogen	Inhibition of Notch cleavage by γ-secretase	[127]	Nausea, vomiting, withdrawal bleeding (in females)	[121, 128]
RO4929097	γ-Secretase-inhibitors	[129]	N/A, clinical trials	[121]

**Table 2:** Reactive oxygen species inhibitors

Compound	Mechanism of action	References	Side effects	SE references
Tiron (4,5-dihydroxy-1,3-benzenedisulfonic acid)	ROS scavenger	[130, 131]	N/A	–
Tempol (4-hydroxy-2,2,6,6-tetramethylpiperydine-1-oxyl)	Superoxide dismutase mimetic, antioxidant	[130, 132]	N/A	–
Glutathione (GSH)	Antioxidant, reduces disulfide bonds	[133]	N/A, clinical trials	[121]
*N*-acetyl-cysteine (NAC)	Antioxidant, breaks disulfide bonds	[133]	Reproductive toxicity, reduction in fertility	[121, 134]
Vitamin C (ascorbic acid)	Antioxidant, reducing agent	[133]	Nausea, vomiting, diarrhea, flushing of the face, headache, fatigue, and disturbed sleep	[135]
Vitamin E (tocopherol)	Fat-soluble antioxidant	[133]	No significant side effects	[136, 137]

**Table 3:** Apoptosis inhibitors

Compound	Mechanism of action	References	Side effects	SE references
BBMP [5-(benzylsulfonyl)-4-bromo-2-methyl-3(2H)-yridazinone]	Mitochondrial permeability transition pore (PTP) inhibitor neuroprotective	[138]	N/A	–
BI-6c9	Bid inhibitor, prevented loss of mitochondrial outer membrane potential (MOMP) and mitochondrial fission	[139–141]	N/A	–
BTZO-1 (1,3-benzothiazin-4-one derivative)	Activation of antioxidant response element (ARE)-mediated gene expression	[142]	N/A	–
Bongkrekic acid solution	Inhibitors of mitochondrial permeability transition (MPT) pore opening	[143–148]	N/A	–
Pifithrin-α hydrobromide	p53 Inhibitor	[149]	N/A	–
NS3694	Inhibitor of apoptosome formation	[150]	N/A	–
Z-ATAD-FMK	Inhibitor of apoptosome formation	[150]	N/A	–
*N*-benzylisatin sulfonamide analogues	Caspase-3 inhibitor	[151]	N/A	–
5-Dialkylaminosulfonylisatins	Caspase-3 and 7 inhibitor	[152]	N/A	–
16F16	Protein disulfide isomerase (PDI) inhibitor	[153]	N/A	–
16F16A	Protein disulfide isomerase (PDI) inhibitor	[153]	N/A	–
16F16A-DC	Protein disulfide isomerase (PDI) inhibitor	[153]	N/A	–
Thiomuscimol	Protein disulfide isomerase (PDI) inhibitor	[153]	N/A	–
Cystamine	Protein disulfide isomerase (PDI) inhibitor	[153]	Loss of appetite, diarrhea, drowsiness, lack of energy, nausea, stomach pain, unpleasant breath odor, vomiting	[154, 155]
Pifithrin-α	p53 Inhibitor	[156, 157]	N/A	–
Pifithrin-μ (PFTmu, 1)	Inhibits p53 binding to mitochondria	[158]	N/A	–
S-15176 difumarate salt	Inhibits mitochondrial permeability transition	[159]	N/A	–
IDN-6556	Inhibitor of caspase 3	[160]	N/A	–

**Table 4:** Autophagy activators

Compound	Mechanism of action	References	Side effects	SE references
10-(6′-Ubiquinonyl)decyltriphenylphosphonium (MitoQ)	Redox-active ubiquinone that accumulates in mitochondria	[161]	N/A, clinical trial	[121]
1-Alpha, 25-dihydroxy vitamin D3	Ca++ regulator	[162]	Hypercalcemia	[121, 163]
	VDR modulator			
2-Deoxyglucose (2-DG)	Binds to hexokinase, marker for tissue glucose use	[164, 165]	N/A, clinical trials	[121]
5-Fluorouracil	Inhibits thymidylate synthase	[166]	Nausea, vomiting, diarrhea, gastrointestinal ulceration and bleeding, and bone marrow depression	[121, 167]
6-Hydroxydopamine	Neurotransmitter	[168]	N/A	[121]
A23187	ER stress	[169]	N/A	[121]
Amiodarone	Anti-arrhythmic drug	[170]	Abnormal taste or smell, constipation, decreased sexual interest, dizziness, dry eyes, flushing of the face, general body discomfort, headache, loss of appetite, nausea, tiredness, trouble sleeping, vomiting	[121, 171]
Ammonia	By-product of amino acid metabolism	[172]	N/A	[121]
Arsenic trioxide	MEK/ERK pathway	[173, 174]	QT interval prolongation, arrhythmia, tachycardia, fatigue, fever, edema, convulsions, muscle weakness, confusion	[121, 175]
	Beclin 1 or Atg7 targets			
	Induces XCP, not XCA			
Aspirin	Inhibitor of mTOR and activator of AMP-activated protein kinase	[176]	Black, bloody, or tarry stools, coughing up blood or vomit, nausea, stomach pain, fever, upset stomach, heartburn, headache, tinnitus	[121, 177]
Atorvastatin	AMP-activated protein kinase	[178]	Muscle pain, memory problems, fever, unusual tiredness, dark-colored urine, swelling, thirst, dry mouth, nausea	[121, 179]
Aurintricarboxylic acid (ATA)	ERK1/2 activation	[180]	Adverse effect for focal nodular hyperplasia	Information from the manufacturer
AZD-8055	mTOR ATP-competitive inhibitor	[181]	N/A, clinical trials	[121]
Benzaldehyde	Formation of autophagosome	[182]	N/A, clinical trials	[121]
Bortezomib	PSMB5 inhibitor, 26S proteasome inhibitor	[183, 184]	Nerve problems, dry cough, trouble breathing, headache, vision problems, bleeding, fever, fast or slow heart rate, nausea, diarrhea	[121, 185]
Brefeldin A	Endoplasmic reticulum (ER)-to-golgi traffic inhibitor	[186]	N/A	–
Ca2+	Via Ca++ channels	ref in [187, 188]	N/A	–
Capsaicin	Through the AMPKα-mTOR signaling pathway and the accumulation of p53 in the nucleus	[189]	Nausea, vomiting, abdominal pain burning diarrhea, eye exposure	[190]
Carbamazepine (CBZ)	Radiation protector and mitigator	[191, 192]	Dizziness, drowsiness, dry mouth, nausea, unsteadiness, vomiting	[121, 193]
	Anti-epilepsy and mood-stabilizing drug			
Ceramide	Protein kinase B inhibition	[194, 195]	N/A	–
Cetuximab	EGFR inhibitor	[196]	Changes in fingernails or toenails, constipation, cough, diarrhea, dry mouth, headache, indigestion, nausea, pain, swelling, stomach pain or upset, vomiting, weakness, weight loss	[121, 197]
Cisplatin	DNA-damaging agent	[198]	Diarrhea, loss of appetite, nausea, vomiting, weakness	[121, 199]
Chlorpromazine	Used to treat the symptoms of schizophrenia	[200]	Agitation, constipation, dizziness, drowsiness, dry mouth, enlarged pupils, jitteriness, nausea, stuffy nose	[121, 201]
Cholecalciferol	Vitamin D	[202]	Constipation	[121, 203]
Clonidine	G(i) signaling activator	[204]	Anxiety, confusion, constipation, dizziness, drowsiness, dry mouth, general weakness, nausea, ringing in the ears, sweating, tiredness, vomiting	[121, 205]
Cucurbitacin	STAT3 inhibitor	[206]	N/A	–
Deforolimus, Ridaforolimus	MTOR inhibitor	[207]	Tiredness and feeling weak, loss of appetite, sore mouth and throat, rash, a drop in blood cells causing an increased risk of infection, tiredness and breathlessness, diarrhea, fatigue, anorexia	[205, 208]
Delta-9-tetrahydrocannabinol (THC)	Psychoactive ingredient in marijuana	[209]	CNS adverse	Information from the manufacturer
Dexamethasone	Glucocorticoid hormones	[210]	Problems with vision, swelling, pancreatitis, depression, bloody stools, low potassium, high blood pressure	[121, 211]
Digeranyl bisphosphonate	Inhibitor of geranylgeranyl diphosphate synthase (GGDPS)	[212]	Renal toxicity, acute-phase reactions, gastrointestinal toxicity, and osteonecrosis of the jaw	[213]
d-Glucose	ER stress	[165]	N/A, clinical trials	[121]
Docosahexaenoic acid (DHA)	Through p53/AMPK/mTOR signaling	[214]	Back pain, flu, infection, pain, angina pectoris, dyspepsia, eructation, rash, taste perversion	[121, 215]
Doxorubicin	Antitumor antibiotic, chemotherapy drug	[216]	Cardiac failure, arrhythmias, thrombocytopenia, leukopenia, nausea, vomiting, ulceration in the gastrointestinal tract, hyperuricemia, nephropathy	[217]
Epothilone B	Microtubule-stabilizing agent	[218]	N/A, clinical trials	[121]
Esomeprazole magnesium	Proton-pump inhibitor	[219]	Headache, diarrhea, abdominal pain	Information from the manufacturer
Etoposide	Topoisomerase inhibitor	[220]	Loss of appetite, back pain, blue or purple discoloration of the skin, diarrhea, hair loss, increased sweating, nausea, tightness in throat, voice changes, vomiting	[121, 221]
Everolimus	MTOR inhibitor; FKBP1A binder	[222]	Stomatitis, rash, diarrhea, fatigue, edema, abdominal pain, nausea, fever, andheadache	[223]
Fluspirilene	Diphenylbutylpiperidine antipsychotic drug	[200, 224]	Sleepiness, movement disorders, pain where the injection is given	[225, 226]
GNE477	Dual PI3K/mTOR inhibitors	[181]	N/A	–
Glucose-6-phosphate	Glucose homeostasis	[227]	N/A	–
Gossypol	Natural phenol BCL2 inhibitor	[228]	N/A, clinical trials	[121]
GSK2126458	Dual PI3K/mTOR inhibitors	[181]	N/A, clinical trials	[121]
Hydrogen peroxide	Reactive oxygen species, signaling molecule	[229]	Nausea and vomiting, burns in the mouth, throat, esophagus, and stomach, bleeding in the stomach, inflammation of the intestines	Information from the manufacturer
INK 128	mTOR ATP-competitive inhibitor	[181]	N/A, clinical trials	[121]
Imatinib	PDGFRA, PDGFRB inhibitor	[230]	Nausea, vomiting, diarrhea, loss of appetite, dry skin, hair loss, swelling, and muscle cramps	[121, 231]
	BCR/ABL tyrosine kinase inhibitors			
Ionomycin	Potent a Ca++ ionophore	[232]	Changes in lung function, i.e., pneumoconiosis	Information from the manufacturer
Lactacystin	Enhanced degradation of aggregated proteins	[187]	N/A	–
Lanosterol	Regulates mitochondrial function	[233]	Changes in lung function, i.e., pneumoconiosis	Information from the manufacturer
l-Arginine	Amino acid	[234]	N/A, clinical trials	[121]
Leupeptin	Protease inhibitor	[235]	N/A	–
Lipopolysaccharide (LPS)	Bacterial endotoxin	[236]	LPS is an adjuvant for both humoral and cell-mediated immunity. It augments the immune response to both protein and polysaccharide antigens. It is too toxic and pyrogenic, even in minute doses, to be used as an adjuvant in humans	Information from the manufacturer
Lithium, lithium chloride	Treatment of bipolar disdorders	[237]	Tremor, muscle hypertonicity, ataxia, choreoathetotic movements, cardiac arrhythmia, hypotension, peripheral circulatory collapse, anorexia, nausea, vomiting, gastritis	[121]
Loperamide	Opioid-receptor agonist	[200]	Constipation, drowsiness and nausea	[121, 238]
LY294002—morpholine derivative of quercetin	PI3K inhibitor	[198]	N/A, preclinical	[121]
Melphalan	Reversible inhibitor of PI3Ks DNA-damaging drug	[239]	Vomiting, ulceration of the mouth, diarrhea, and hemorrhage of the gastrointestinal tract	[121, 240]
Mesoridazine		[200]	Emesis, muscle tremors, decreased food intake	Information from the manufacturer
Metformin	Activator of AMP-activated protein kinas (AMPK)	[176, 241]	Epigastric discomfort, nausea, and vomiting, diarrhea, drowsiness, weakness, dizziness, malaise, and headache	[121, 242]
Minoxidil	K + ATP channel opener	[204]	Rapid heartbeat, faintness, dizziness, chest pain, nausea, sweating, trouble breathing, easy bruising or bleeding	[121, 243]
Modified Yeoldahanso-tang	Neuroprotection	[187]	N/A	–
Nelfinavir	HIV protease inhibitors (HIV PIs)	[244]	Diarrhea, gas, loss of appetite, nausea, stomach pain	[121, 245]
Niclosamide	Anthelmintic	[170]	Abdominal pain, anorexia, diarrhea, and emesis	[246]
Niguldipine	Ca++ channel blocker	[200]	Discontinued	[121]
Nortriptyline	Tricyclic antidepressant	[200]	Card dizziness, drowsiness, dry mouth, headache, impotence, nausea, pupil dilation, sensitivity to sunlight, sweating, upset stomach, vomiting, weakness, weight loss	[121, 247]
OSI-027	MTOR ATP-competitive inhibitors	[181]	N/A	–
Oxidative stress		Ref in [187]	N/A	–
Oxaliplatin	Inhibiting phosphorylation of mTOR	[248]	Constipation, decreased appetite, diarrhea, dizziness, fatigue, gas, hair loss, headache, heartburn, hiccups, increased tears, mild stomach pain, muscle or joint aches, nausea, runny nose, taste changes, trouble sleeping, vomiting, weight loss	[121, 249]
Pepstatin A	Inhibitors of cathepsin proteases B and D	[212]	N/A	–
Perifosine	Akt inhibitor	[250]	Nausea, fatigue, vomiting, diarrhea, anorexia	[121, 251]
Perhexiline	Inhibiting mitochondrial carnitine palmitoyltransferase-1	[170]	Nausea, transient dizziness, hypoglycemia in diabetic patients	[121, 252]
PI-103	Dual PI3K/mTOR inhibitors	[181]	N/A, preclinical	[121]
N(10)-substituted phenoxazine (10-NCP)	Akt inhibitor	[200]	N/A	–
Phorbol myristate acetate (PMA)	Protein kinase C (PKC) activator	[253]	N/A, clinical trials	[121]
Phosphatidylinositol-3-phosphate	Membrane phospholipid	[254]	N/A	–
Pimozide	Antipsychotic drug of the diphenylbutylpiperidine class	[200]	Constipation, drowsiness, dry mouth, restlessness	[255]
Promazine	Phenothiazine class of antipsychotics	[200]	Extrapyramidal symptoms, drowsiness, weight gain, dry mouth, constipation, endocrine effects, hemolytic anemia	[121, 256]
Promethazine	Antihistamine of the phenothiazine family	[200]	Mild depression of CNS and cardiovascular system, profound hypotension, respiratory depression, unconsciousness	[121, 257]
Propranolol	Cardiodepressant	[258]	Bradycardia, cardiac failure, hypotension, and bronchospasm	[121, 259]
Rapamycin (Sirolimus)	Antibiotic	[181, 260]	N/A, clinical trials	[121]
Reactive oxygen species	DNA damage	[206]	N/A	–_
Resveratrol	Nature phytoalexin	[261]	N/A, clinical trials	[121]
Rilmenidine	Anti-hypertensive drug	[67]	Asthenia, palpitations, insomnia, drowsiness, fatigue on exercise, epigastric pain, dryness of the mouth, diarrhea, skin rash	[262]
Rottlerin	Kinase inhibitor	[170]	N/A	–
Saquinavir	Antiretroviral protease inhibitors	[263]	Anxiety, blurred vision, body fat changes, changes in sexual desire, constipation, diarrhea, dizziness, dry lips or skin, gas, headache, heartburn, mouth sores, nausea, night sweats, sleeplessness, stomach discomfort, taste changes, tenderness or bleeding of the gums, tiredness, vomiting, warts, weight gain	[121, 264]
Sodium selenite	Activation of DAPK via PP2A-mediated dephosphorylation at Ser(308)	[265]	N/A, clinical trials	[121]
Sorafenib	Multikinase inhibitor	[266]	Diarrhea and dermatologic events	[121, 267]
Spermidine	Inhibition of mTOR or activation of AMPK	[261, 268]	N/A	–
Staurosporine	Kinase inhibitor	[269]	N/A	–
Superoxide	Oxidative stress	[270]	N/A	–
Tamoxifen	Anti-estrogen	[271]	Hot flashes, hypercalcemia, peripheral edema, distaste for food, pruritus vulvae, depression, dizziness, lightheadedness, headache	[121, 272]
Temsirolimus	MTOR binder	[271]	Hypersensitivity, hyperglycemia, interstitial lung disease, hyperlipidemia, bowel perforation, renal failure	[121, 273]
Thapsigargin	Non-competitive inhibitor of sarco/endoplasmic reticulum Ca++ ATPase	[232]	N/A, preclinical	[121]
Thioguanine	Nucleoside analog induces DNA mismatch repair	[274]	Nausea, vomiting, malaise, hypotension, and diaphoresis	[2, 275]
Thioridazine	Antipsychotic drug belonging to the phenothiazine drug	[200]	Agitation, blurred vision, confusion, constipation, difficulty breathing, dilated or constricted pupils, diminished flow of urine, dry mouth, dry skin, excessively high or low body temperature, extremely low blood pressure, fluid in the lungs, heart abnormalities, inability to urinate, intestinal blockage, nasal congestion, restlessness, sedation, seizures, shock	[121, 276]
Trehalose	Natural disaccharide implicated in anhydrobiosis	[277]	N/A, clinical trials	[121]
Tretinoin	Retinoic acid	[68, 69]	Headache, fever, weakness, and fatigue	[121, 278]
Triflupromazine	Antipsychotic medication of the phenothiazine class	[200]	Agitation, convulsions, difficulty breathing, difficulty swallowing, dry mouth, extreme sleepiness, fever, intestinal blockage, irregular heart rate, low blood pressure, and restlessness	Information from the manufacturer
UCN-01 (7-hydrostaurosporine)	Akt inhibitor	[279]	N/A	–
Valinomycin	K(+)-selective ionophore	[280]	N/A	–
Valproic acid	Anti-epilepsy and mood-stabilizing drug	[192]	Constipation, diarrhea, dizziness, drowsiness, headache, increased or decreased appetite, mild hair loss, nausea, sore throat, stomach pain or upset, trouble sleeping, vomiting, weakness, weight gain	[121, 281]
Verapamil	Cardiodepressant	[258]	Chest pain, arrhythmia, heart attacks, significant water retention, dizziness	[282]
Vitamin K2 (menaquinone-4)	Vitamin	[70]	N/A, clinical trials	[121]
Vorinostat	HDAC6 inhibitor	[283]	Diarrhea, nausea, anorexia, weight decrease, vomiting, constipation, thrombocytopenia, anemia	[121, 284]
VX-680	Aurora-B kinase inhibitor	[285]	N/A, clinical trials	[121]
WJD008	Dual PI3K/mTOR inhibitors	[181]	N/A	–
Y 27632	ROCK inhibitors	[286]	Discontinued for hypertension	[121]
zVAD	Pan-caspase inhibitor	[287, 288]	N/A	–
Zoledronate	Inhibitor of farnesyl diphosphate synthase (FDPS)	[212]	Hypocalcemia, hypophosphatemia, hypomagnesemia	[121, 289]
68093	ND	[290]	N/A	–
169676	Eg5 inhibitor possibly	[290]	N/A	–
175493	ND	[290]	N/A	–
363998	ND	[290]	N/A	–
4-Piperidinone	Mitotic inhibition	[290]	N/A	–
Aatiram	ND	[290]	Adverse effect for allergic contact dermatitis	[121] Information from the manufacturer
Acridine Yellow	DNA damage	[290]	N/A	–
Bafilomycin A1	Vacuolar ATPase inhibitor	[290]	N/A	–
Bepridil	Ca++ channels	[290]	Dizziness, lightheadedness, diarrhea, heartburn, nausea, blurred vision, muscle cramps, headache, fatigue, drowsiness, ringing in the ears, flushing, trembling, or shaking hands	[291]
Diosgenin	BK Ca++ channel	[290]	N/A	–
E6 Berbamine	Calmodulin inhibitor	[290]	N/A	–
Fluspiriline	Potassium channels	[290]	Gynecomastia, impotence, agranulocytosis, galactorrhea, tachycardia, blurred vision, pyrexia, cataracts, dyskinesia	[225]
Loperamide	Ca++ channels	[290]	Constipation, decreased urination, red, swollen, blistered, or peeling skin, stomach bloating, swelling, or pain	[121, 238]
Monensin	Na+ ionophore	[290]	Adverse effect for myoglobinuria	[121] Information from the manufacturer
Nigericin	Induces intracellular acidification	[290]	N/A	–
Purpurine	ND	[290]	N/A	–
Pyridine derivative	ND	[290]	N/A	–
Rottlerin	PKC delta inhibitor	[290]	Suppressed cell/tissue growth or development	
Stannane	Aquaporin inhibitor	[290]	N/A	–
Tetrandrine	Ca++ channels	[290]	Immunosuppressant activity	[292]
Tetrocarcin A	BCL-2 inhibitor/ER stress	[290]	N/A	–
Thalicarpine	p-Glycoprotein inhibitor/DNA damage	[290]	N/A	–
Trichostatin-A	Histone deacetylase inhibitor	[290]	N/A, clinical trials	[121]
Trifluoperazine	Calmodulin inhibitor possibly	[290]	Agitation, constipation, dizziness, drowsiness, dry mouth, enlarged pupils, headache, jitteriness, loss of appetite, nausea, stuffy nose, tiredness	[293]
Tyrphostine 9	PDGF-R tyrosine kinase inhibitor	[290]	N/A	–

**Table 5:** Methyl transferase inhibitors

Compound	Mechanism of action	References	Side effects	SE references
5-Azacytidine (Vidaza)	Nucleoside inhibitors—[DNMT] enzyme trapping and degradation	[294]	Nausea, anemia, thrombocytopenia, vomiting, fever, diarrhea, neutropenia	[295]
5-Azadeoxycytidine (decitabine)	Nucleoside inhibitors—[DNMT] enzyme trapping and degradation	[294]	Constipation, cough, diarrhea, dizziness, hair loss, headache, joint or muscle pain, loss of appetite, nausea, stomach pain or upset, trouble sleeping, vomiting	[296]
Zebularine	Nucleoside inhibitors	[294]	N/A	–
Procaine	Mask DNMT target sequences	[294]	Chest pain or slow, irregular heartbeats, dizziness, anxiety, nausea, convulsions	[297]
Epigallocatechin-3-gallate (EGCG)	Green tea’s active ingredient	[294]	Nausea and indigestion, neural tube defect	[298]
RG108	DNA methyltransferase inhibitor	[294]	N/A	–
Procainamide	Drug for treatment of cardiac arrhytmia, non-covalent inhibitor of Na + channel	[299]	Rash, myalgia, fever. Treatment with procainamide can cause antibody production against cellular components, accounting for the systemic lupus erythematosus-like adverse reactions	[300]
Parthenolide	Modulation of NF-kappa β activity, microtubule interfering	[301]	Vomiting, abdominal pain, and indigestion	[302]
Curcumin	Natural phenol, gives yellow color to turmeric, interferes with NF-kappa β, mTOR inhibitor	[303]	Mild nausea or diarrhea, iron deficiency	[304]
MithramycinA	Antineoplastic antibiotic, RNA synthesis inhibitor	[305]	Changes in lung function, reduction in the number of white blood cells and platelets and bleeding	[306]
NSC 14778	Non-covalent inhibitor with a new scaffold	[307]	N/A	–
Nanaomycin A	Antifungal antibiotic, DNMT3B inhibitor	[308]	N/A	–

## Targeting *Lamin A* and *Progerin* expression via signal transduction pathways

On the basis of the computational analysis outlined above, a number of potential targets and therapeutic interventions have been identified and discussed. Many of these potential interventions may nonspecifically down-regulate the level of expression of both *Lamin A* and its disease-associated allelic variants, while others have more specific effects on mutant *Lamin A* expression.

### Restoration of IGF-1 and GH balance

Insulin-like growth factor 1 (IGF-1) signaling is involved in aging and longevity in many animals, including nematodes, *Drosophila,* and mammals (for review see [43]). Zmpste24 (−/−) mice, a mouse model of progeria, exhibits dysregulation of somatotropic axis, characterized by high levels of circulating growth hormone (GH) and reductions in insulin-like growth factor-1 (IGF-1) [44]. Application of recombinant IGF-1 restores the balance between IGF-1 and GH, and this delays the onset of several progeroid characters and prolongs the lifespan of progeroid animals [44, 45]. However, applying such an approach as a means of treating HGPS may be limited, given the diverse biological effects exerted by this hormone, and in particular, the pathogenic role of IGF-1 signaling in cancer [46].

### Notch signaling inhibitors

Expression of *Progerin* ectopically activates effectors of Notch and downregulates the canonical Wnt signaling pathway, regulating the differentiation of mesenchymal stem cells [47, 48]. This leads to misregulation of somatic stem cell differentiation, explaining some of the pathological defects of HGPS [49]. Thus, inhibitors of Notch signaling (Table 1) and recombinant β-catenin could potentially ameliorate symptoms of HGPS. Notch 2 may also be targeted for inhibition given its influence on *Granzyme B* transcription [50] and potentially apoptosis; however, the impact of such an approach on immune function is unclear. Furthermore, there are severe side effects known to be associated with certain Notch inhibitors, including gastrointestinal bleeding and skin cancer [51]. Therefore, caution must be taken when applying such approaches to treating HGPS patients.

### Reactive oxygen species scavengers

Basal levels of reactive oxygen species (ROS) as well as induced levels of H_2_O_2_ are five times higher in HGPS fibroblasts compared to normal fibroblasts, which leads to double stranded breaks (DSBs) in DNA and a decrease in the proliferative capacity of cells [52]. Indeed, HGPS is accompanied by an elevated quantity of DSBs and attenuation of their repair [53]. On the contrary, the ROS scavenger *N*-acetyl cysteine (NAC) has been shown to decrease basal levels of DSBs and enhance population-doubling times in fibroblasts derived from HGPS patients [52]. Other effective ROS scavengers are listed in Table 2. However, the effectiveness of using ROS scavengers for treating progeria patients and ameliorating intracellular damage and associated symptoms remains somewhat speculative. In addition, the complex biochemical effects of some anti-oxidants may raise some safety concerns. While the anti-oxidative effects of ascorbic acid are well characterized, pro-oxidative effects have also been described [54–56] and such properties must be taken into consideration when developing treatments for progeria.

### Telomerase activators

Progressive attrition of telomeres causes activation of progerin production in normal human fibroblasts [9]. Active telomerase prolongs the cellular lifespan of HGPS by decreasing progerin-induced DNA-damage signaling and activation of both the p53 and Rb pathways. These are the two pathways that mediate the onset of premature senescence in HGPS [57]. Telomerases can be stimulated by a potent telomerase activator, TA-65, to extend short telomeres, and this has been shown to have a positive effect on health span in mice [58]. Treatment with TA-65 may prevent some symptoms of HGPS. However, while the results in mice are promising, the use of this technology in humans may be limited, particularly in the event of any significant side effects being identified in future clinical trials.

### Rb regulators

Progerin accumulation leads to premature replicative cellular senescence [59]. Marji et al. suggest, based on global gene profiling of HGPS fibroblasts, that defects in the lamin A-Rb signaling pathway may be key factors in the accelerated aging phenotype of HGPS, and perhaps in normal aging, too [60]. Rb activity can be modified with reagents such as roscovitine and PD-0332991, inhibitors of Cdk2–cyclin E and Cdk4/cyclin D1 complexes, respectively, that phosphorylate and inactivate the Rb tumor suppressor [61]. However, studies indicate that Rb expression is decreased in fibroblasts in both HGPS and normal aging, with a concomitant reduction in phosphorylation [60, 62]. In this respect, an intervention that increases Rb expression and/or increases Rb phosphorylation to normal physiological levels may provide some therapeutic benefit in HGPS.

### Apoptosis inhibitors

Nuclear progerin accumulation leads to accelerated aging and increased apoptosis in individuals suffering from HGPS [59], and the same observation has been made in aging HGPS fibroblasts [63]. Theoretically, apoptosis inhibition (Table 3) may offer some means of extending the cellular lifespan of HGPS patients. However, the potential for increased risk of developing cancers and other related diseases would almost certainly limit such an approach.

### Inhibitors of translation and autophagy activators

Rapamycin, an immunosuppressant drug, delays cellular senescence and organismal aging, abrogates nuclear blebbing, and stimulates degradation of progerin in HGPS cells [64, 65]. This drug can selectively decrease progerin levels in progeria cells through a mechanism involving autophagic degradation [66]. Rapamycin treatment decreases the formation of nonsoluble aggregates of progerin and induces progerin elimination by autophagy in normal fibroblasts [64]. A safer alternative to rapamycin, rilmenidine, a centrally acting anti-hypertensive drug, was found to induce autophagy in cell culture via a pathway independent of the mammalian target of rapamycin [67]. As a natural alternative to the acid form of tretinoin (all-trans-retinoic acid), vitamin A has been found to induce autophagy. The essential oil produced from rose hip seeds is a natural source of tretinoin and promotes autophagosome maturation through a pathway independent from the classic nuclear hormone receptors [68, 69]. Another natural autophagy activator is vitamin K2 [70]. A comprehensive list of known autophagy activators is listed in Table 4.

Several small molecules down-regulate lamin A/C protein via mechanisms of proteolysis. Doxorubicin (also known as adriamycin) is a topoisomerase II inhibitor used in anti-cancer therapy whereby it induces activation of caspases, leading to cleavage of lamin A/C [71]. Doxorubicin has also been identified in a high-content screen for inducers of autophagy [72]. Sangivamycin is a nucleoside analog that acts via activation of JNK and protein kinase C delta. In MCF-7/Adr cells, sangivamycin increases cleavage of human lamin A/C protein [73]. Tunicamycin and thapsigargin, endoplasmatic reticulum (ER) stress inducers, increase degradation of mouse LMNA protein via the activation of caspases [74]. Paclitaxel, a chemotherapeutic agent, induces cleavage of lamin A/C, enhanced by the synthetic peptides Smac/DIABLO [75]. It should be noted that HGPS is characterized by a large increase in the rate of apoptosis [63], and while application of apoptosis inducers in HGPS treatment is restricted, autophagy inducers can be considered safe, since they do not induce cell death [72].

### cAMP activators

Several hormones have been identified as having effects on the expression, function, or phosphorylation of *Lamin A*. Hormones that increase cAMP levels (glucagon, calcitonin, vasopressin, and parathyroid hormone (PTH)) decrease lamin A protein phosphorylation of rat LMNA protein in renal medullary thick ascending limb cells [76]. cAMP-dependent phosphorylation controls nuclear lamin associations, and aberrant phosphorylation could cause remodeling of the lamina [77]. Therefore, hormone modulation of lamin A phosphorylation with glucagon, calcitonin, vasopressin, or parathyroid hormone might be another way to alleviate laminopathy symptoms.

### Thyroid hormone supplementation

Thyroid hormone (T3) decreases expression of mouse *Lamin A* mRNA in liver from mice exhibiting hypothyroidism [78]. An association has been reported between low levels of T3 and DeBarsi syndrome, an autosomal recessive syndrome characterized by a progeria-like appearance [79, 80]. The endocrine system is affected by aging, and while T3 has been associated with longevity, deficiencies in, or suboptimal levels of T3 are more common in older individuals, particularly women. Therefore, supplementation with T3 might have beneficial effects on progeria symptoms, as well as aging [81, 82].

#### PI3K pathway inhibitors

In T98G cells stimulated with the growth factor PDGF, inhibition with a small molecule inhibitor of PI3K (LY294002), which prevents Akt phosphorylation, has been found to decrease expression of human *LMNA* mRNA induced by the PDGF-BB. This demonstrates that *LMNA* may be regulated through the phosphatidylinositol 3-kinase PI3K pathway [83]. It has been suggested that aberrant phosphorylation of Ser458 of *Lamin A* by Akt1 contributes to striated muscle laminopathies caused by *LMNA* mutation [84]. Therefore, inhibition of Akt1 by LY294002 might be beneficial to this and similar laminopathies.

### Epigenetics marks reversal

Reversal of epigenetic marks may represent a novel anti-aging target. In an animal model of progeria, Zmpste24-deficient mice show hypermethylation and transcriptional silencing of rDNA genes. This effect is reversible through treatment with methyltransferase inhibitors [85]. Therefore, methyltransferase inhibitors (Table 5) could prevent HGPS symptoms. In the same animal model it has been noted that a delayed DNA damage response is a result of histone H4 acetylation defect. Reversal of this defect by supplying the histone deacetylase inhibitor sodium butyrate in drinking water ameliorated aging-associated effects, and extended the lifespan in the animal model. In addition to accumulation of progerin, aged mice show hypoacetylation of the histone H4K16 [86]. Therefore, treatment with methyltransferase inhibitors (Table 5) and histone deacetylase inhibitors could potentially reduce HGPS symptoms. However, such interventions could give rise to significant side effects and would have to be carefully evaluated and refined before transfer to the clinical setting.

### Targeting posttranslational modification: farnesylation inhibitors

Ionafarnib (SCH-66336), a farnesyltransferase inhibitor, has been shown to inhibit prelamin A farnesylation in buccal mucosa cells [87]. Other studies demonstrate that inhibitors of farnesyltransferase (FTIs) ameliorate the phenotype of transgenic G608G *LMNA* mice [88]. This model is characterized by extensive and progressive loss of vascular smooth muscle cells (VSMCs) of large arterial media [89], similar to effects observed in human HGPS patients [90, 91]. FTIs have also been shown to improve survival and bone integrity in LMNA HG/+ [92, 93] and in ZMPSTE24−/− mouse models [94]. The compound FTI-277 may completely restore localization of nuclear antigens in HGPS fibroblasts [59]. The combination of statins and aminobisphosphonates has been shown to inhibit the production of farnesylation and geranylgeranylation modifications of prelamin A and progerin in Face-1/Zmpste24-defective mice, decreasing senescence-like symptoms and increasing the lifespan of affected mice [95, 96]. Unfortunately, the FTI treatment has harmful side effects such as centrosome separation and bipolar spindle formation defects, nuclear dysmorphy, and cytotoxicity [97]. In addition, mice, expressing nonfarnesylated progerin variants (LMNA(nHG/+)), still reveal progeria-like phenotypes, which are not ameliorated by FTI [98].

## Directly and selectively targeting mutant *Lamin A*

### Targeting mutant *Lamin A* RNA: antisense oligonucleotides, RNAi, miRNA, and siRNA

Inhibition of the LMNA miss-spliced site reverses senescence-associated defects in cell nuclei [20]. Fong et al. demonstrated the effectiveness of antisense oligonucleotide technology and identified an antisense oligonucleotide which is complementary to a site in exon 11 at a 5′ position relative to the alternate splice site in LMNA transcripts. This may be used to decrease alternative splicing in HGPS fibroblasts and moderately reduce progerin levels [99]. Splicing-based therapeutical approaches have been examined using a genetically modified mouse strain that carries an HGPS mutation. Antisense morpholino-based therapy has been developed with the aim of preventing pathogenic LMNA splicing, and alleviating the progeroid phenotype [100].

For the past decade, cancer treatments have been developed based on RNA interference (RNAi)—a mechanism that effectively “shuts down” malfunctioning genes with small noncoding RNA molecules from the families of microRNAs. LMNA transcripts have a myriad of microRNAs with which they specifically interact; hence, an RNAi approach offers potential for targeting misspliced LMNA transcripts. In the brain, the *Prelamin A* transcript is regulated by the brain-specific microRNA miR-9. The tight shutdown of the LMNA transcript observed in the brain using miR-9 may explain the lack of central nervous system pathology in mouse models of HGPS [101]. Recently, Weidefield et al. have reported on the generation of a conditional inducible microRNA (RNAi) system for the controlled inactivation of LMNA [102]. There is also evidence of differential expression of miRNAs in control versus LMNA-related laminopathy [103]. A systemic application of siRNA, specifically targeted to tissues of interest may offer promising potential in future therapeutic applications.

### Targeting mutant protein accumulation: chemical protein binding

A proteomics approach using matrix-assisted laser-desorption-ionization time of flight (MALDI-TOF) MS [104] has identified that lamin A belongs to a family of poly (ADP-ribose) binding proteins. Nuclear lamin A was found to be covalently bound to acetaminophen (APAP), and also appears to become phosphorylated upon arylation. Lamin A may be associated with disruption of nuclear membrane organization, which may be triggered by the translocation of the 55- to 58-kDa APAP-protein adduct, leading to cell death [105]. Direct lamin A/chemical binding may be explored by designing a molecular sponge that sequesters mutant lamin A, i.e., progerin from the cell.

### Gene therapy: nanotherapy, viral vectors, protocells, and targeting progerin for autophagy

Several gene therapies and nanotherapies targeting cellular proteins are currently in development. One such approach to the aggregation of misfolded proteins has been applied in the case of a Hungtinton’s neurodegeneration (HD) mouse model (HDR6/1) by targeting proteins for autophagic proteosomal degradation using intrabodies. This may represent an effective strategy if modified for clearance of progerin instead [106]. Genome customization and targeted gene modification of *Lamin A* mutant alleles using gene-specific engineered nucleases such as zinc finger nucleases or transcription activator-like effector nucleases (TALENs, Cellectis) represents another possible approach [107].

The assortment of gene delivery vehicles for gene therapy products are expanding, and include lentivirals and adeno-associated viral vectors [108] in addition to non-viral gene delivery systems such as lipoplexes, polyplexes, inorganic nanoparticles, quantum dots, and protocells [109–111]. However, despite significant progress in recent years, limitations still persist in refining this technology for use in the clinical setting both in terms of patient safety and efficacy.

## Future directions

The role of lamin A is to maintain nuclear structure and integrity and in doing so, contribute to the health and survival potential of individuals within a population. The consequences of defectiveness in lamin A structure and function are observed, therefore, to be extremely severe, manifesting at several broad-ranging and essential biological levels, which include cell signaling and gene expression. As such, any intervention successful in ensuring the maintenance of LMNA protein function and/or the reduction of LMNA’s downstream effects must work within key parameters at each of these regulatory levels. Our analysis has identified potential targets for therapeutic intervention by addressing both causes and effects of LMNA defectiveness. Our findings provide a framework for targeting LMNA defectiveness directly at the genetic level and further downstream by targeting signaling events and other processes which give rise to cellular insult and ultimately disease.

**Fig. 2:**
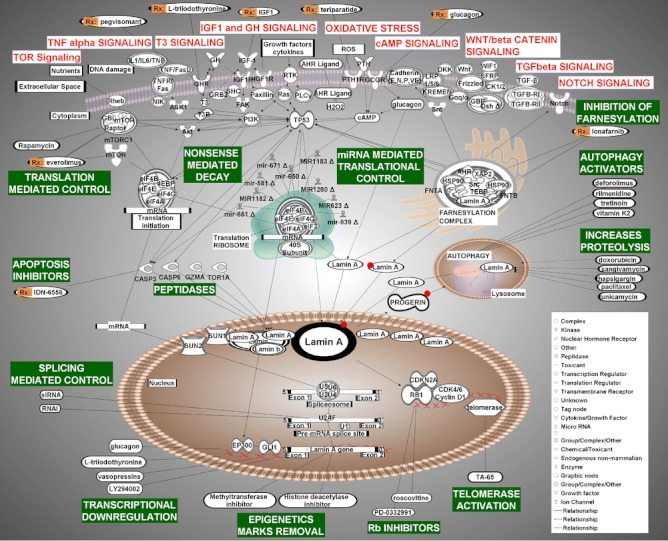
Presented are steps of the gene expression cycle of *Lamin A*/*C* that can be targeted for additional regulation: transcription, splicing, translation, posttranslational modification, and degradation via autophagy. See text for a description for strategies for targeting each of these steps with already-available drugs to minimize the deleterious effects of altered *Lamin A* expression in laminopathies, and possibly aging. Gene names and *symbols* are the same as those listed in Fig. 1. *Red dots* represent farnesylation residues on lamin A/progerin

By means of a computational analysis of multiple biological pathways, we have identified a number of plausible therapeutic targets, and outlined 12 possible interventions for regulating defective LMNA expression and protein accumulation (see Fig. 2). These are: (1) IGF-1 and GH balance restorers, (2) Notch signaling inhibitors, (3) reactive oxygen species scavengers, (4) telomerase activators, (5) Rb inhibitors, (6) apoptosis inhibitors, (7) translation- and autophagy-activator inhibitors, (8) cAMP activators, (9) thyroid hormone supplementation, (10) PI3K pathway inhibitors, (11) epigenetics marks reversal, and (12) farnesylation inhibitors. While these targets are highly specific in many cases, collectively they are wide ranging and cover the biological complexities that characterize LMNA-related diseases and the levels at which they manifest. Furthermore, we have presented a comprehensive list of compounds known to act on specific targets within these biologic pathways. A combinatory approach may be applied using this data to develop a therapy or therapies consisting of a combination of several key compounds, potentially including: farnesylation inhibitors, autophagy activators, apoptosis inhibitors, and telomerase activators.

Extensive in vitro studies characterizing the effectiveness of these compounds are required before moving towards translation into clinical practice. In particular, the effects of these compounds on progerin accumulation is of particular interest and may be used as a measure of treatment efficacy; in part, it could also be used in the validation of such interventions. However, there are several outstanding questions that must be answered in order to validate the effectiveness of such interventions and the mechanisms by which they provide benefit. Outstanding questions in this field concern the molecular mechanisms by which laminopathies and related diseases manifest and the processes in which progerin alters cellular phenotype and biological age.

In addition to identifying molecular targets, the approach outlined here has also focused on identifying molecules that can carry out specific functions. The use of small molecules to activate/inhibit the signal transduction pathways that regulate LMNA expression itself represents one such approach. In this manner, lamin A in addition to progerin may be down-regulated to levels that effectively influence cellular homeostasis to a point at which cells are healthy, producing lower levels of both laminA and progerin, and therefore potentially lowering the rate at which aging occurs. There are many small molecules/drugs presently available and approved by the FDA (albeit for other purposes), which may be used to target signaling pathways in this way (Tables 1, 2, 3, 4 and 5). However, as part of this strategy, it will be necessary to further characterize the significance of the ratio of progerin/laminA in disease pathogenesis. A long-term goal may be to develop a method of directly targeting of the underlying causes of progeria and related conditions. This requires a technological platform that targets and discriminates progerin and other products of disease-causing *Lamin A* alleles, from functional lamin A. There are potential tools available at present that may be tailored further for this purpose. In particular, it is feasible that progerin may be specifically targeted at the mRNA level using RNAi tools. In order to target toxicity associated with mutant protein accumulation, designer proteases may also be developed to specifically degrade the mutant protein. In addition, the use of intrabodies to bind and target progerin for autophagy also represents a potential means for achieving these aims. Delivery mechanisms will also be key to any such therapeutic intervention, and the use of viral vectors and nanotherapeutic delivery approaches hold much promise as they are developed and refined into the future. Antisense morpholino-based gene therapy also holds much promise. By directing this technique to prevent pathogenic LMNA splicing, Osorio et al. have achieved reductions in progerin accumulation and associated nuclear defects, amelioration of progeroid phenotypes, and an extension of lifespan [100]. While the gene therapy approach requires further refinement, this technology clearly represents the most likely means of ensuring correct splicing and localization of defective LMNA. Drugs which enhance autophagic mechanisms to achieve reductions in progerin accumulation also show considerable potential; however, safer analogues are required [112]. Another approach may be the use of small molecules to ameliorate the effects of progerin accumulation, such as those listed in this review. However promising, further evaluation of the potential impact of these molecules on the disease phenotype are required prior to application in the clinical setting, either individually or in combination with other therapies. Currently, a number of clinical trials are underway to examine the potential therapeutic effects of using statins, FTIs, and bisphosphonate in combination to treat progeria [113, 114]. Similarly, some of the molecules highlighted in this study may be incorporated into future clinical trials or treatments.

The overall focus of this review has been to identify and highlight different methods that may be used for treating laminopathies, and to a lesser extent, other LMNA-associated human diseases and aging. While some treatments may act to target the downstream effects of progerin accumulation, other treatments may be used to directly alter the ratio of progerin/wild-type protein. Indeed, the most effective method of treating laminopathies would be to target and counteract progerin accumulation directly. However, the targeting of *LMNA* expression in general also holds potential for treating patients given that disease severity may be determined in part by the ratio of progerin to mature lamin A. In order for future treatments to significantly alter the ratio of wild-type/mutant protein in favor of cellular health and longevity, mechanisms that achieve increments comparable to wild-type protein levels may be required in addition to also reducing progerin levels. Modulating the expression of *Lamin A* may also be effecive for the treatment to other human diseases associated with *LMNA*, given that differential allele-specific expression has been identified at the *LMNA* locus [40]. For example, alleles represented at relatively high frequencies in human populations have been associated with a number of relatively common human diseases (e.g., rs4641). In this respect, development of methods of increasing expression of wild-type LMNA may offer a means of treating both laminopathies and other human diseases association with genetic variation at this locus.

The consequences to intervening to alter the ratio of *Progerin*/*LMNA* expression are likely to be significantly influenced by a host of factors, including underlying differences in cell-type and tissues to which treatment is directed. In particular, *LMNA* expression is known to be developmentally regulated, being expressed in differentiated cells while being absent from early embryonic stem cell compartments and at low to negligible levels in hematopoietic systems [115–117]. This points to the importance of *LMNA* expression in the maintenance of the differentiated cell state [115]. In HGPS, it has been suggested that stem-cell-driven tissue regeneration may be reduced and tissue-specific differences in apoptosis or regenerative potential may give rise to the tissue-specific segmental aging pattern [118]. Any treatment developed to therapeutically alter the *Progerin*/*LMNA* ratio should therefore consider the potential for distinctive role(s) for *Lamin A* in different tissue compartments.

## Conclusions

In conclusion, our analysis describes a range of potential therapeutic approaches that may be used to modulate the expression and accumulation of defective lamin A and/or modify its downstream effects in laminopathies and age-related diseases. However, careful evaluations of these approaches and the potential side effects of drug treatments discussed here are required before consideration as therapeutic treatments in a clinical setting.
